# Investigation of CO_2_ Absorption Rate in Gas/Liquid Membrane Contactors with Inserting 3D Printing Mini-Channel Turbulence Promoters

**DOI:** 10.3390/membranes13120899

**Published:** 2023-12-04

**Authors:** Chii-Dong Ho, Luke Chen, Jr-Wei Tu, Yu-Chen Lin, Jun-Wei Lim, Zheng-Zhong Chen

**Affiliations:** 1 Department of Chemical and Materials Engineering, Tamkang University, Tamsui, New Taipei 251301, Taiwan610400110@gms.tku.edu.tw (Y.-C.L.); 611400598@o365.tku.edu.tw (Z.-Z.C.); 2 Department of Water Resources and Environmental Engineering, Tamkang University, Tamsui, New Taipei 251301, Taiwan; luke@mail.tku.edu.tw; 3 HICoE-Centre for Biofuel and Biochemical Research, Institute of Self-Sustainable Building, Department of Fundamental and Applied Sciences, Universiti Teknologi PETRONAS, Seri Iskandar 32610, Perak Darul Ridzuan, Malaysia; junwei.lim@utp.edu.my; 4 Centre for Herbal Pharmacology and Environmental Sustainability, Chettinad Hospital and Research Institute, Chettinad Academy of Research and Education, Kelambakkam 603103, Tamil Nadu, India

**Keywords:** carbon dioxide absorption, 3D mini-channel turbulence promoter, absorption flux improvement, Sherwood number, concentration polarization effect

## Abstract

The CO_2_ absorption by Monoethanolamine (MEA) solutions as chemical absorption was conducted in the membrane gas absorption module with inserting 3D mini-channel turbulence promoters of the present work. A mathematical modeling of CO_2_ absorption flux was analyzed by using the chemical absorption theory based on mass-transfer resistances in series. The membrane absorption module with embedding 3D mini-channel turbulence promoters in the current study indicated that the CO_2_ absorption rate improvement is achieved due to the diminishing concentration polarization effect nearby the membrane surfaces. A simplified regression equation of the average Sherwood number was correlated to express the enhanced mass-transfer coefficient of the CO_2_ absorption. The experimental results and theoretical predictions showed that the absorption flux improvement was significantly improved with implementing 3D mini-channel turbulence promoters. The experimental results of CO_2_ absorption fluxes were performed in good agreement with the theoretical predictions in aqueous MEA solutions. A further absorption flux enhancement up to 30.56% was accomplished as compared to the results in the previous work, which the module was inserted the promoter without mini channels. The influences of the MEA absorbent flow rates and inlet CO_2_ concentrations on the absorption flux and absorption flux improvement are also illustrated under both concurrent- and countercurrent-flow operations.

## 1. Introduction

The absorption rate of conventional contactors with chemical absorbents is restricted because the operational limitations of liquid channeling, flooding, entrainment, and foaming [1], which was overcome by membrane contactors with combining techniques of conventional separation technology and the presence of the membrane. The membrane contactors are membrane-based separation processes [2] such as membrane extraction [3], membrane absorption [4], ion exchange membrane [5] and membrane distillation [6] offers the advantageous features of low energy consumption, large and stable gas-liquid contact area, continuous operations, modulation arrangement and easy up-scaling [7]. Membrane absorption is one of the membrane contactors applied to the undesirable gas removal such as CO_2_ and H_2_S from the gas mixture for reducing greenhouse gas emission in industrial processes. The CO_2_ absorption in conventional contactors with chemical absorbents is promoted and studied widely in the decade years [8] by several technologies namely membrane absorption [9], membrane adsorption [10] and membrane processes [11] as a promising technology with a higher absorption efficiency. The advantage of a higher specific area would be beneficial to absorption efficiency at the expense in the membrane contactor of an additional mass transfer resistance due to the membrane’s presence [12]. Membrane absorption is the one that the non-wetted microporous hydrophobic membrane serves as a barrier separating CO_2_ gas feed stream and absorbent stream; the gas/liquid interface was formed at the membrane pore mouth in the gas feed stream. Moreover, Monoethanolamine (MEA) as an amine absorbent [13] has been used at high pressures [14] in the alkanolamine-based CO_2_ membrane absorption process. Both chemical reaction and physical absorption occur simultaneously due to gas diffusing through the membrane into the liquid phase [15,16], as confirmed by the previous study [17] according to the diffusion-reaction model [18,19]. In addition, the selective membrane materials [20] were durable and reusable [21,22] as well as the properties of absorbents [23] that examined the device performance of a successful process intensification for CO_2_ absorption processes. Comprehensive understanding of the mass transfer mechanism of the CO_2_ absorption rate [24] was developed with Knudsen-molecular diffusion of the dusty gas model [25] to estimate the mass flux [26,27] transporting through the membrane.

The mass-transfer boundary layers adjacent to the membrane surface results in the reduction of the concentration driving-force gradient as well as the absorption flux. The concentration polarization effect [28] plays an important role in deteriorating the concentration gradient, which leads to the decrement of transmembrane flux in the membrane contactor module, and thus the absorption rate is decreased [29]. Several aspects of influencing the concentration polarization effect were assessed such as the hydrodynamic conditions and feed concentration. The absorption efficiency was augmented by inserting turbulence promoters such as spacer filaments [30] and carbon-fiber spacers [31] to diminish the concentration polarization effect, which come out with a higher convective mass-transfer coefficient due to increasing the turbulence intensity [32]. An effective strategy was investigated to capture CO_2_ in turbulent flow patterns [33] instead of operating a laminar flow velocity of absorbent feed stream. Turbulence intensity could be enhanced near the membrane surface with the use of eddy promoters to disrupt the mass-transfer boundary layer as well as the concentration polarization reduction. The magnitude of the concentration polarization coefficient $\gamma_{m}$ is an indicator to evaluate the device performance of the membrane absorption module. The larger the value of $\gamma_{m}$ that is obtained, the higher the mass transfer flux of CO_2_ transports from the gas side to the absorbent feed stream. Moreover, an alternative configuration of reducing the turbulent boundary layer region [34] was proposed by using carbon-fiber spacers [35] into the flowing channel with avoiding overly exceptional power consumption. A higher CO_2_ absorption rate enhancement at the expense of power consumption due to destroying the viscous laminar sublayer adjacent to the membrane interface was taken into account the economic consideration. A new design proposed the membrane absorption module with embedding 3D mini-channel turbulence promoters in the MEA feed channel to improve a higher CO_2_ absorption efficiency in the present study.

Various amines and mixed amines [13] were used widely in chemical absorption technology for many decades to enhance the CO_2_ capture rate and to attain regeneration cost down [36] as well. The one-dimensional modeling of mass-balance and chemical reaction equations was successfully developed and formulated with occurring reaction mechanisms of CO_2_ absorption [37]. MEA absorbents [38] were used to improve the CO_2_ absorption flux in the hydrophobic microporous membrane contactor system [37]. This paper examines the effects of an increasing shear rate to disturb the concentration boundary layer by implementing 3D mini-channel turbulence promoters in flat-plate membrane contactors, and to perform theoretical predictions and experimental results of the CO_2_ absorption flux in parallel-plate gas/liquid PTFE/PP (polytetrafluoroethylene/polypropylene) membrane contactors with the use of MEA as an absorbent. The 3D printing technology presents a higher flexibility [39] in precisely tailoring and fabricating various complex 3D geometric shapes of turbulence promoters [40] to develop various hydrodynamic conditions. Membrane filaments were commonly employed in membrane separation modules to create eddy currents and flow disruption, which lead to the diminished concentration polarization effect and enhanced permeate flux. The influences of turbulence intensity amplification were accomplished by embedding 3D printing mini-channel turbulence promoters in the MEA feed stream, which were incorporated and regressed into the dimensionless quantities called mass-transfer enhancement factors under both cocurrent-flow and countercurrent-flow operations, respectively.

Microscale devices are a promising area of process intensification that could generate both technological and economic benefits [41]. The turbulence intensity of laminar flows is increased when the fluid flows forward in the mini-channel, thereby improving the mass transfer rate of the synergy between velocity field and concentration field [42]. Meanwhile, the ratio of the modified Sherwood number of turbulent flow to the Sherwood number under laminar flow was correlated in terms of various parameters such as geometric shapes of turbulence promoters, flow configurations, operation types, inlet concentrations and MEA feed flow rates. The new design achieves a considerable CO_2_ absorption flux enhancement by embedding 3D printing mini-channel turbulence promoters in the MEA feed channel as compared to conducting the module with using an empty channel. Moreover, the CO_2_ absorption flux improvement associated with a higher Sherwood number and the trade-off of power consumption increment was also delineated by considering the economic sense on both module designs and system operations. Actually, this study extends the previous study [43] to the membrane absorption module instead of inserting mini-channel turbulence promoters in obtaining a higher absorption efficiency and absorption flux improvement. The purpose of the present study is to discuss the effects of the geometric shapes of turbulence promoters, array configurations, flow patterns, inlet CO_2_ concentrations and MEA feed flow rates on the device performance in a flat-plate membrane absorption module with inserting mini-channel turbulence promoters.

## 2. Experimental Setup

**Figure 1:**
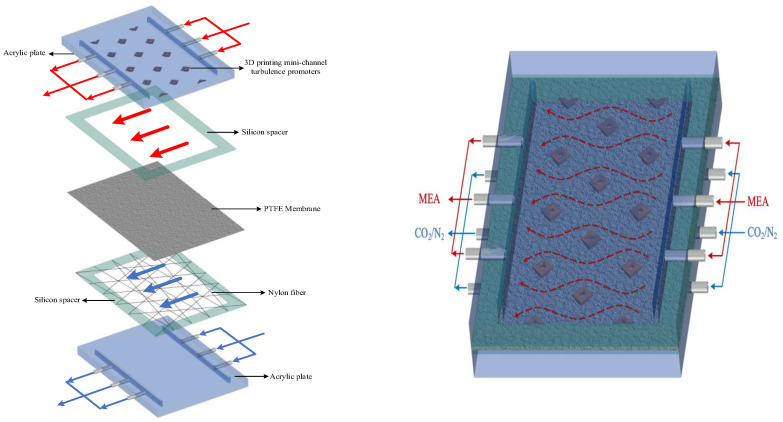
Fabrication structure of components in a flat-plate membrane contactor module.

The fabrication structure of a flat-plate membrane contactor module for CO_2_ absorption by the MEA absorbent with implementing 3D mini-channel turbulence promoter is illustrated in Figure 1 with flow paths indicated in red and blue dash lines and arrows.

**Figure 2:**
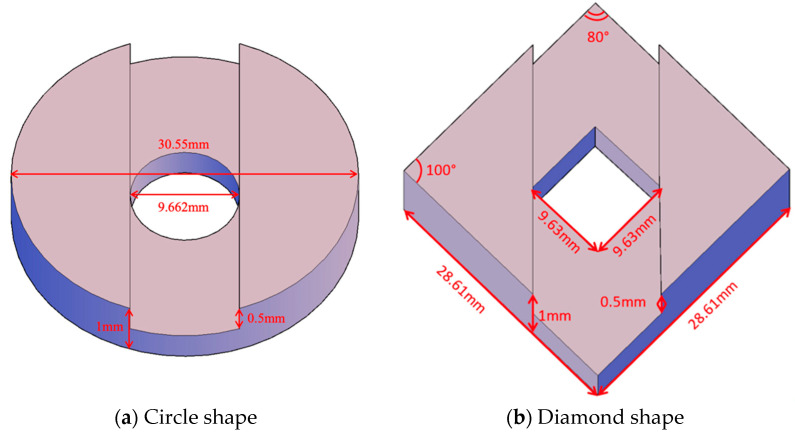
Front view of two shapes of 3D mini-channel turbulence promoters.

**Figure 3:**
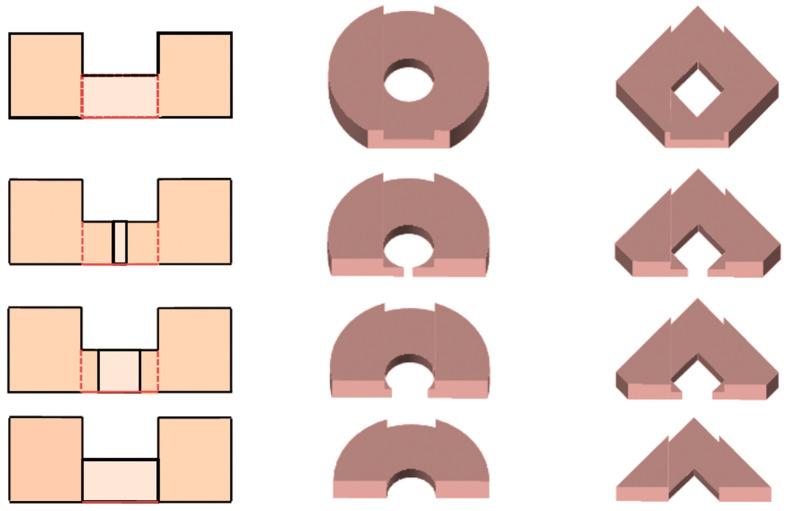
Cross-sectional views of various sectors of 3D mini-channel turbulence promoters.

The printing material of turbulence promoters was made with polyester elastomer and stuck onto the hydrophobic membrane surface. The average molecular weight of the polyester elastomer (Polylactic Acid, PLA) ranges between 1000 and 60,000 with density of 1180 kg/m^3^. Two shapes of turbulence promoters were fabricated with 1 mm height by a 3D printer (ATOM 2.5EX, Hsinchu County, Taiwan), say Circle and Dimond types, and inserted in the flowing channel for conducting experiments. The detail of the 3D printing protocol of the fabricated turbulence promoters of two geometric shapes with dimensions, say Circle and Diamond shapes, and cross-sectional views of various sectors were embedded into MEA absorbent flow channel, as shown in Figure 2 and Figure 3, respectively. The Circle type was made of a diameter of 30.00 mm and the Diamond type was made of 28.61 mm with each length, which the printing promoter icon hindering permeate passages and deteriorating gas permeate flux due to turbulence promoter coverage of the membrane surface area.

**Figure 4:**
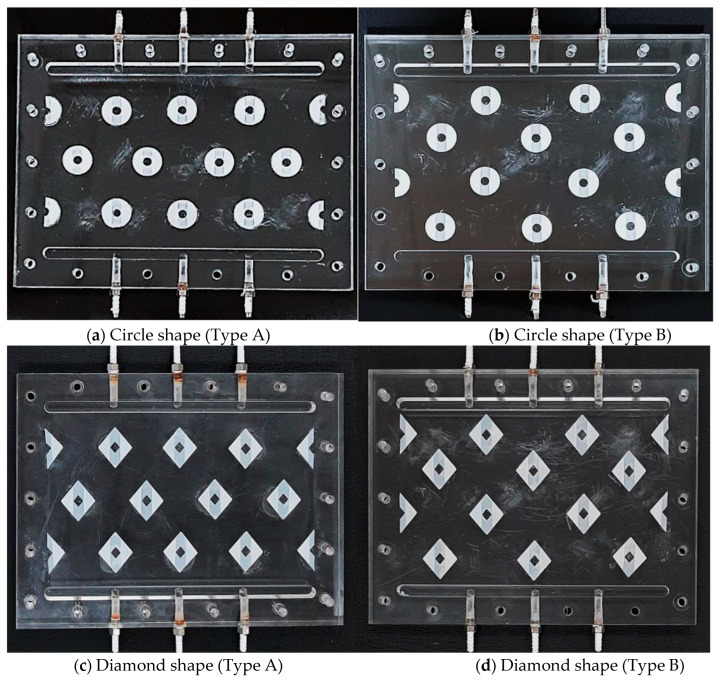
Photos of two shapes and two configurations of 3D mini-channel turbulence promoters.

Two array configurations were arranged, say Type A and Type B, as shown in Figure 4. The 3D printing technology presents a higher flexibility in designing various complex geometric shapes of turbulence promoters in precisely tailoring through a layer-by-layer machining process by a 3D printer (ATOM 2.5EX, Mastech Machine Co., Ltd., New Taipei, Taiwan). Those turbulence promoters were manufactured and submerged into the MEA solution with a total durability test time of 48 h to ensure that they are resistant and stable to corrosion before conducting the experiments runs. Meanwhile, about 13% occupation of the printing turbulence promoter icons on the membrane surface was counted the effective permeate flux area in the calculation procedure due to blocking permeate flux through the membrane. The flat-plate membrane contactor module contains two flow channels with embedding 3D mini-channel turbulence promoters onto the MEA absorbent feed stream, and the other empty channel for CO_2_/N_2_ gas mixture with winding nylon fiber of 0.2 mm diameter upon the hydrophobic membrane surface as a supporting material to prevent from vibration and wrinkling. Two 1 mm-thick silicon rubbers were sealed between the hydrophobic composite membrane and the acrylic plate for both absorbent and gas feed sides, respectively, to build up flow channels and to prevent leakage. The 3D printing turbulence promoters of 1 mm-thick were fabricated the mini-channel flow path and glued with Cyanoacrylate Adhesive (Chang Chun Plastics Co., Ltd., Taipei, Taiwan) on the acrylic plate of the MEA absorbent feed side in contact with the hydrophobic membrane surface to create the eddy motion around those impediments. A parallel conduit (*L* = 0.21 m, *W* = 0.29 m, *H* = 2.0 mm) to conduct two flow channels separated by inserting a hydrophobic composite membrane made of PTFE/PP (ADVANTEC) as the permeating medium with a nominal pore size of 0.2 µm, a porosity of 0.72, and a total thickness of 130 µm (PTFE 98 µm and PP 32 µm). 

**Figure 5:**
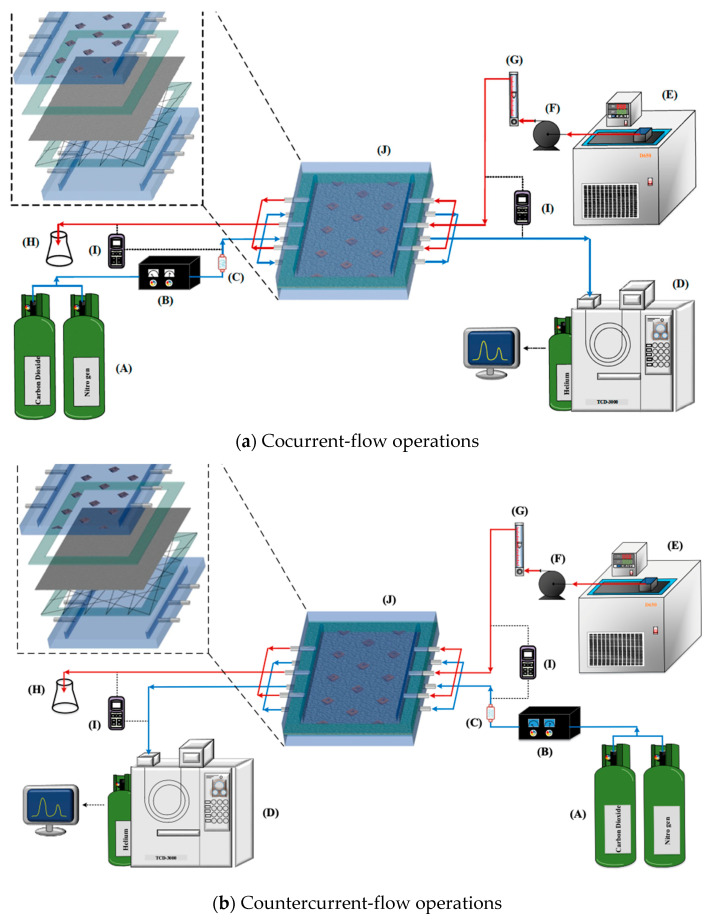
Schematic representation of flat-plate gas membrane absorption systems. (**A**) Gas cylinder; (**B**) Gas mass flow controller; (**C**) Gas mix adapter; (**D**) Gas chromatograph; (**E**) Constant temperature water tank; (**F**) Pump; (**G**) Flowmeter; (**H**) Erlenmeyer flask; (**I**) Thermometer; (**J**) Membrane absorption module.

The experiments were conducted for controlling various 30 wt% MEA ($5.0\times 10^{3}$ mol/m^3^) MEA absorbent feed flow rates (5.0, 6.67, 8.33, 10.0 cm^3^/s) regulated by a flow meter (MB15GH-4-1, Fong-Jei, New Taipei, Taiwan) as the liquid absorbent pumping from a reservoir. Meanwhile, a gas mixture containing CO_2/_N_2_ was pumped from the gas mixing tank (EW-06065-02, Cole Parmer Company, Vernon Hills, IL, USA) by using the mass flow controller (N12031501PC-540, Protec, Brooks Instrument, Hatfield, PA, USA) at 5 cm^3/^s with various CO_2_ concentrations of 30%, 35% and 40%, respectively. The schematic detailed configuration of flat-plate membrane contactor modules for CO_2_ absorption by MEA absorbent are illustrated in Figure 5 under both cocurrent- and countercurrent-flow operations.

**Figure 6:**
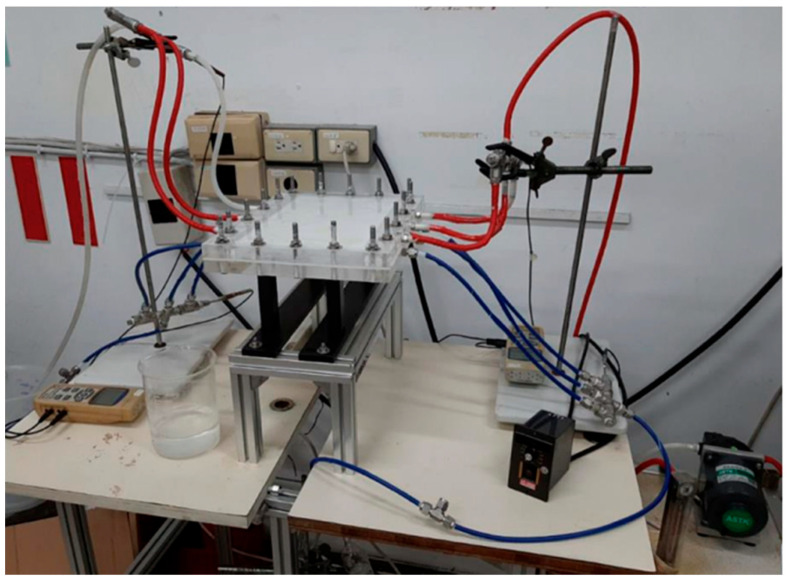
A photo of an experimental apparatus of a flat-plate gas membrane absorption system.

A photo of the operating experimental setup of a flat-plate gas membrane absorption system is shown in Figure 6 with acrylic plates as outside walls. Comparisons were made of CO_2_ absorption flux under various operation conditions between the flat-plate membrane contactor modules with/without inserting 3D printing mini-channel turbulence promoters. The outlet CO_2_ sample was collected and injected into the column heating systems for rapid heating of the sample-collection capillary tube, which was measured by using the gas chromatography with helium as a carrier gas (Model HY 3000, China Chromatograph Co., Ltd., Xinzhuang, New Taipei, Taiwan) to analyze the CO_2_ concentrations including conventional thermal conductivity detector (TCD) devices.

## 3. Mathematical Treatments

### 3.1. Concentration Polarization 

**Figure 7:**
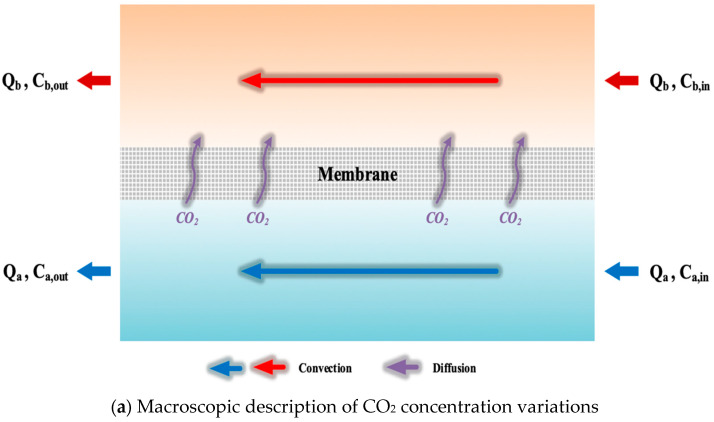

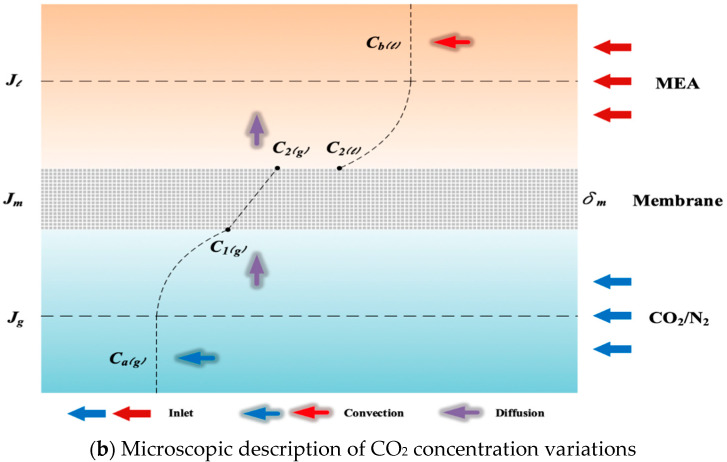
Schematic mass transfer resistances and concentration profiles of membrane contactor.

The concentration at the membrane surface affecting the CO_2_ concentration driving-force gradient across the membrane, consequently influencing the absorption flux. A mass-transfer behavior analysis is studied to describe the concentration gradient between both membrane surfaces of CO_2_/N_2_ gas feed side and MEA absorbent feed side. A representation of the mass-transfer behavior in the membrane gas/liquid contactor module is depicted in Figure 7a,b of macroscopic description and microscopic descriptions, respectively. 

$C_{1(g)}$ and $C_{2(g)}\;$ indicate the membrane surface concentrations of the CO_2_/N_2_ gas feed stream and MEA absorbent feed stream, respectively, as seen from Figure 7b. The concentration differences between both streams near the membrane surfaces and those of the bulk stream are used to estimate the temperature polarization coefficient.

Concentration polarization is enumerated by the ratio of the concentration difference across the membrane to the concentration difference of both bulk streams, as is called the concentration polarization coefficient is Equation (1):

(1) $$\gamma_{m}=\frac{C_{1\left(g\right)}-C_{2\left(g\right)}}{C_{a\left(g\right)}-C_{b\left(g\right)}}=\frac{\left(C_{1\left(g\right)}-\frac{{K^{\prime }}_{ex}C_{2\left(l\right)}}{H_{c}}\right)}{C_{a\left(g\right)}-\frac{C_{b\left(l\right)}}{H_{c}}}$$

Declining concentration gradients between the bulk streams and membrane surfaces, as seen in Figure 7b, results in decreasing the mass-transfer driving force, and thus, the CO_2_ absorption flux is reduced. Mass transfer in a membrane gas absorption process occurs within three regions according to the schematic diagram of the membrane gas/liquid contactor module: (i) the CO_2_/N_2_ feed stream; (ii) the microporous hydrophobic membrane and (iii) the MEA absorbent feed stream. The influence of the absorption flux is dominated by the concentration difference for each mass transfer region, which may be represented in Equations (2)–(4) under the steady-state operation as follows:

(2) $$J_{g}=k_{a}\left(C_{a\left(g\right)}-C_{1\left(g\right)}\right)$$

(3) $$J_{l}=k_{b}\left(\frac{{K^{\prime }}_{ex}C_{2\left(l\right)}}{H_{c}}-\frac{C_{b\left(l\right)}}{H_{c}}\right)$$

(4) $$\begin{array}{ll} J_{m} & =c_{m}(P_{1}-P_{2})\frac{1}{M_{w}}={\left.c_{m}\frac{dP}{dC}\;\right|}_{C_{mean}}(C_{1(g)}-C_{2(g)})\frac{1}{M_{w}}=c_{m}RT(C_{1(g)}-\frac{K_{ex}^{\prime }C_{2\left(l\right)}}{H_{c}})\frac{1}{M_{w}}\\ & =K_{m}(C_{1(g)}-\frac{K_{ex}^{\prime }C_{2\left(l\right)}}{H_{c}}) \end{array}$$

The mass flux of CO_2_ diffusing through the trans-membrane was evaluated using the saturation partial pressure differences ($P_{1}-P_{2}$) [44] and membrane permeation coefficient ($c_{m}$) [45] according to dusty gas model [17], in which, $K_{m}$ is the overall mass transfer coefficient of membrane, the CO_2_ concentration on the membrane/liquid interface by using the dimensionless Henry’s law constant $H_{C}=C_{2}/C_{1}=0.73$ [37]. The reduced equilibrium constant at T=298 K [45] and the membrane permeation coefficient [46] with the tortuosity τ=1/ε [47] were determined as follows:

(5) $$K_{ex}^{\prime }=K_{ex}[\mathrm{MEA}]/[H^{+}],K_{ex}=[{\mathrm{MEACOO}}^{-}]\;[H^{+}]/[{\mathrm{CO}}_{2}][\mathrm{MEA}]=1.25\times 10^{-5}$$

(6) $$c_{m}={\left(\frac{1}{c_{K}}+\frac{1}{c_{M}}\right)}^{-1}={\left\{{\left[1.064\frac{\varepsilon \;r_{p}}{\tau \delta_{m}}{\left(\frac{M_{w}}{RT_{m}}\right)}^{1/2}\right]}^{-1}+{\left[{\left|Y_{m}\right|}_{ln}\frac{D_{m}\varepsilon }{\delta_{m}\tau }\frac{M_{w}}{RT_{m}}\right]}^{-1}\right\}}^{-1}$$

**Figure 8:**
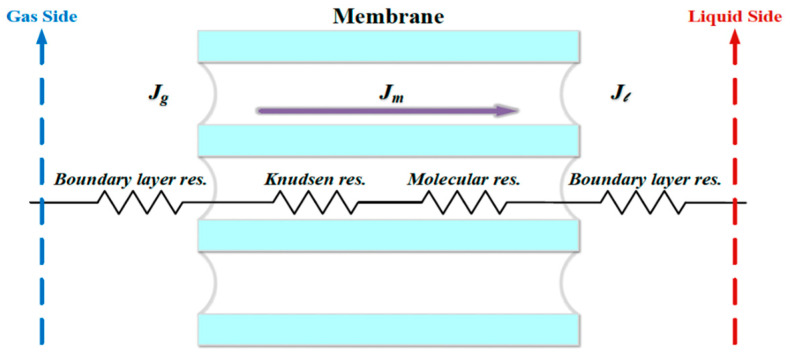
Schematic mass transfer resistances for three mass transfer regions of membrane contactor.

Flux permeating through the CO_2_/N_2_ stream, the microporous hydrophobic membrane and the MEA stream for the modules with/without embedding 3D mini-channel turbulence promoters is shown in Figure 8. Equating the amount of mass flux by the conservation law among three regions, one may obtain the following:

(7) $$J_{i}=J_{g}=J_{m}=J_{l}\;i=promoter,\;empty$$

### 3.2. Concentration Distributions

**Figure 9:**
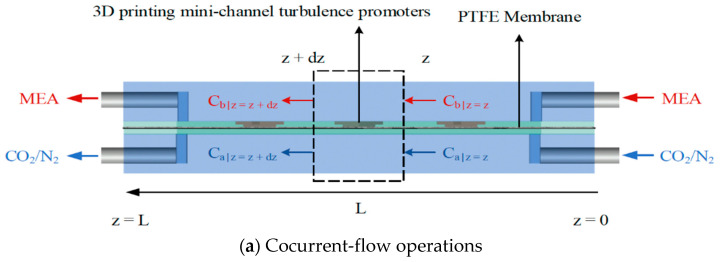

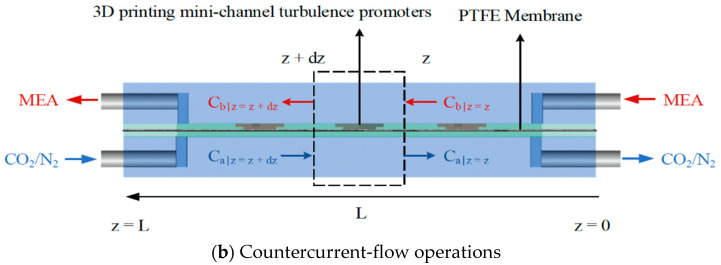
The mass balances made within a finite fluid element.

Balances of mass flux due to mass diffusion and chemical reaction by the mass conservation were formulated simultaneously. The steady-state isothermal diffusion-reaction process in the gas/liquid membrane contactor module causes the trans-membrane mass flux of CO_2_ and were formulated by balancing mass flux conservation presented in a finite control element under concurrent-flow and countercurrent-flow operations in Figure 9a,b, respectively.


(8) $$\frac{dC_{a(g)}}{\mathrm{dz}}=-\frac{W}{Q_{a}}\left[k_{m}\gamma_{m}\left(C_{a(g)}-\frac{C_{b\left(l\right)}}{H_{c}}\right)\right],cocurrent-flow operation$$

(9) $$\frac{dC_{a(g)}}{\mathrm{dz}}=\frac{W}{Q_{a}}\left[k_{m}\gamma_{m}\left(C_{a(g)}-\frac{C_{b\left(l\right)}}{H_{c}}\right)\right],countercurrent-flow operation$$

(10) $$\frac{{\mathrm{dC}}_{b\left(l\right)}}{\mathrm{dz}}=\frac{W}{Q_{b}}\left[k_{m}\gamma_{m}\left(C_{a(g)}-\frac{C_{b\left(l\right)}}{H_{c}}\right)\right]-\frac{k_{{CO}_{2}}C_{b\left(l\right)}\left(\mathrm{WH}\right)}{Q_{b}}$$

in which *z* is the coordinate along with the flowing direction (positive direction), and the concentration polarization coefficient $\gamma_{m}$ was derived and obtained by equating Equations (2) and (4) ($J_{m}=J_{g}$) and Equations (3) and (4) ($J_{m}=J_{l}$), respectively, as follows:

(11) $$C_{a(g)}=C_{1(g)}+\frac{k_{m}}{k_{a}}\left(C_{1(g)}-\frac{{K^{\prime }}_{ex}C_{2\left(l\right)}}{H_{c}}\right)$$

(12) $$\frac{C_{b\left(l\right)}}{H_{c}}=\frac{{K^{\prime }}_{ex}C_{2\left(l\right)}}{H_{c}}-\frac{k_{m}}{k_{b}}\left(C_{1(g)}-\frac{{K^{\prime }}_{ex}C_{2\left(l\right)}}{H_{c}}\right)$$

Then, a simplified form of $\gamma_{m}$ expressed in terms of the mass-transfer coefficient as

(13) $$\gamma_{m}=\frac{\left(C_{1(g)}-\frac{{K^{\prime }}_{ex}C_{2\left(l\right)}}{H_{c}}\right)}{C_{a(g)}-\frac{C_{b\left(l\right)}}{H_{c}}}=\frac{1}{\left(1+\frac{k_{m}}{k_{a}}+\frac{k_{m}}{k_{b}}\right)}=\frac{k_{a}k_{b}}{k_{a}k_{b}+k_{m}k_{b}+k_{m}k_{a}}$$

The procedure for calculating theoretical predictions of the mass transfer coefficient was performed using continuous iterating $C_{1(g)}$ and $C_{2(g)}$ from Equations (11) and (12) within the convergence tolerance. The calculated convective mass-transfer coefficients were delivered to obtain the concentration distributions of both the CO_2_/N_2_ gas feed stream and the MEA absorbent feed stream by solving two simultaneous ordinary differential equations of Equations (8) and (10) for cocurrent-flow operation (or Equations (9) and (10) for countercurrent-flow operation) by marching the fourth-order Runge—Kutta method along the flow direction, as shown in Figure 9.

### 3.3. Mass-Transfer Rate Enhancement

**Figure 10:**
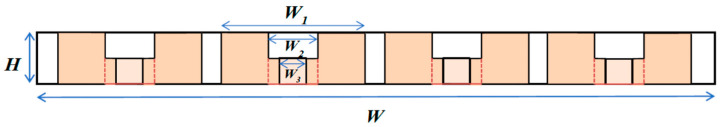
The equivalent diameters of 3D printing mini-channel turbulence promoters.

**Figure 11:**
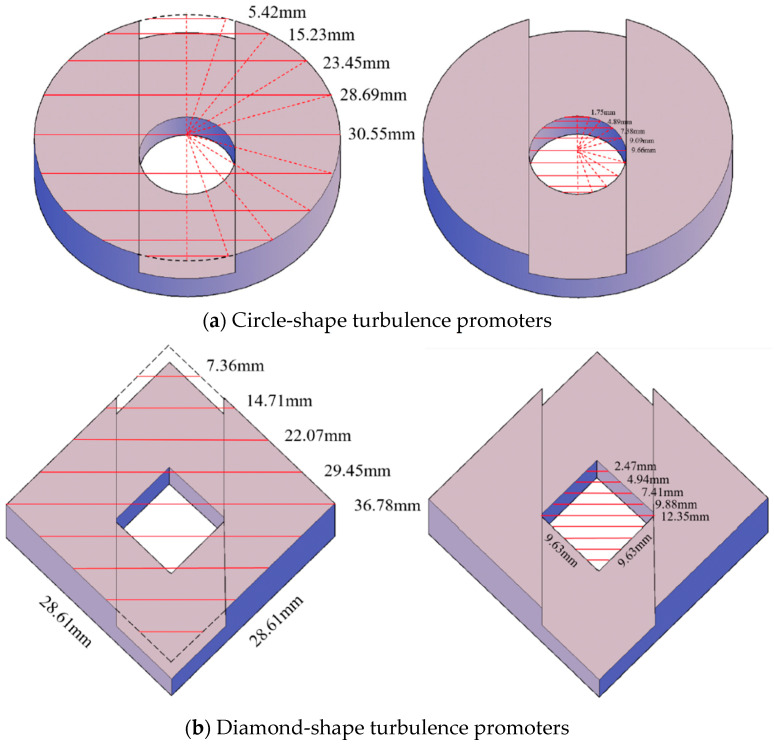
The average diameters of Circle-shape and Diamond-shape turbulence promoters.

The 3D printing mini-channel turbulence promoters are inserted in the conduit of the MEA feed stream instead of using the module of empty channel (without embedding turbulence promoters). The enhancement factor $\alpha^{E}$ depending on the geometric shapes and array configurations was regressed to calculate the enhanced convective mass-transfer coefficients in gas/liquid membrane contactor modules with inserting the 3D printing mini-channel turbulence promoters [33] as follows:

(14) αE=ShpromoterShlaminar=fDh,promoterDh,empty,Re=aDh,promoterDh,emptybRec

where $D_{h,promoter}$ and ${\;D}_{h,empty}$ are the equivalent diameters of modules with embedding 3D mini-channel turbulence promoters and the empty channel in the MEA absorbent feed stream, respectively. The equivalent diameters of modules with embedding 3D turbulence promoters $D_{h,promoter}$ was calctlated by the wetted area A and wetted perimeter P, say 4A/P, as shown in Figure 10. In which, $W_{1}$ is the average width of the promoter, $W_{2}$ is the punched hole diameter designed by one-third promoter diameter, and $W_{3}$ is the the average width of the punched hole inside the promoter. Meanwhile, the average diameters of mini-channel turbulence promoters were estimated by averaging various sections of both Circle and Diamond shapes, as shown in Figure 11.

## 4. Results and Discussions

### 4.1. Flux Improvement by Inserting Min-Channel Turbulence Promoters in Membrane Modules

**Figure 12:**
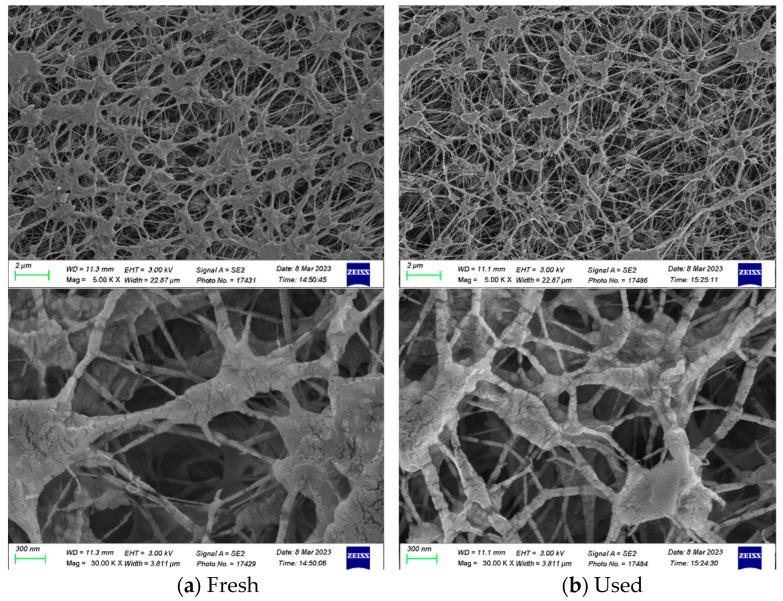
SEM images of the PTFE/PP membrane for fresh and used membranes of experimental runs.

**Figure 13:**
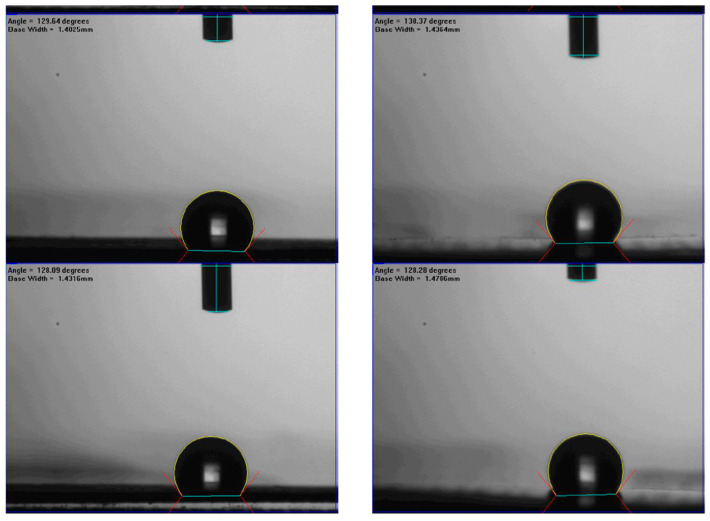
Sessile-drop contact angles of PTFE/PP membranes.

The morphology and water contact angle of the PTFE/PP membranes were characterized by using Scanning Electron Microscopic (SEM, Zeiss sigma 300, Jena, Germany) and Contact angle system (First Ten Angstrom FTA-125, Portsmouth, NH, USA). Figure 12 shows the morphology of the fresh and used membranes of experimental runs. The SEM images were completed by applying a beam of high-energy electrons, which indicated that the presence of the 3D printing mini-channel turbulence promoter of no fouling or scaling in conducting experimental runs. Moreover, the hydrophobic membrane surface wettability can be portrayed with water contact measurements by establishing the tangent (angle) of a liquid drop on a solid surface at the base, which is defined by the mechanical equilibrium of the liquid drop under the action of three interfacial tensions. The water contact angles of the hydrophobic composite membrane made of PTFE/PP were shown in Figure 13. The PTFE/PP membranes presented different surface wettability in the range of 128–132° (water contact angle of 130.3 ± 2.0°) with the confirmation of the surface hydrophobicity of the hydrophobic membrane.

The absorption flux is dependent on the concentration gradients between both membrane surfaces in the gas/liquid membrane contactor modules. The modules with inserting mini-channel turbulence promoters of various geometric shapes and array configurations to diminish concentration polarization effect due to reducing of the mass-transfer boundary-layer thickness. The absorption flux improvement with respect to the MEA feed flow rates and inlet feed CO_2_ concentrations are more substantial in concurrent-flow operations than those in countercurrent-flow operations regarding to the effects of the geometric shapes, array configurations and flow patterns with embedding mini-channel turbulence promoters. The accuracy deviation [48] was calculated between the experimental results and theoretical predictions as follows:

(15) $$Er\;(\%)=\frac{1}{N_{exp}}\sum_{j=1}^{N_{exp}}\frac{\left|J_{theo,j}-J_{\exp,j}\right|}{J_{\exp,j}}\;$$

Moffat [48] determined the experimental uncertainty for each individual measurement from the experimental runs as follows:

(16) $$S_{J_{exp}}={\left\{\sum_{i=1}^{N_{exp}}\frac{{\left(J_{exp,i}-\bar{J_{exp,i}}\right)}^{2}}{N_{exp}-1}\right\}}^{1/2}$$

The mean value of the resulting uncertainty of the experimental measurements was defined by

(17) $$S_{\bar{J_{exp}}}=\frac{S_{J_{exp}}}{\sqrt{N_{exp}}}$$

where *N_exp_*, $J_{\exp,i}$ and $J_{theo,i}$ are the number of experimental data, theoretical predictions and experimental results of absorption fluxes, respectively. The accuracy deviations and mean uncertainty were calculated within $3.2\times 10^{-3}\leq Er\leq 5.23\times 10^{-2}\;$ and $5.21\times 10^{-3}\leq S_{\bar{J_{exp}}}\leq 8.32\times 10^{-3}$ for both cocurrent- and countercurrent-flow operations. The good agreement was expected between the theoretical predictions and experimental results.

**Figure 14:**
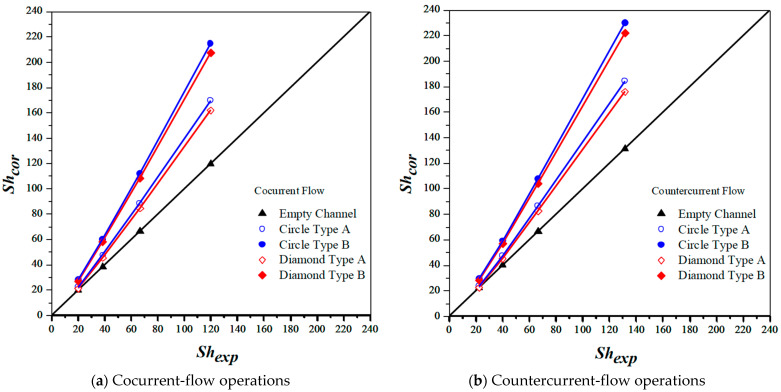
Comparison of correlated and experimental Sherwood numbers for various array configurations of 3D printing min-channel turbulence promoters.

Implementing 3D printing mini-channel turbulence promoters with two geometric shapes of Circle and Diamond and two array configurations produces the augmented turbulence intensity, which results in the higher absorption flux under both cocurrent- and countercurrent-flow operations. The mass transfer coefficients of the module with inserting mini-channel turbulence promoters in the flow channel can be incorporated into the correlated Sherwood number, as referred to Equation (14), and determined by using Buckingham’s π theorem for cocurrent- and countercurrent-flow operations, respectively, as presented in Equations (18) and (19) as well as in Figure 14a,b.

(18) αE=ShpromoterShlaminar=0.596 Dh,promoterDh,empty2.245Re0.351Cocurrent-flow operations

(19) αE=ShpromoterShlaminar=0.488 Dh,promoterDh,empty2.077Re0.413Countercurrent-flow operations

**Figure 15:**
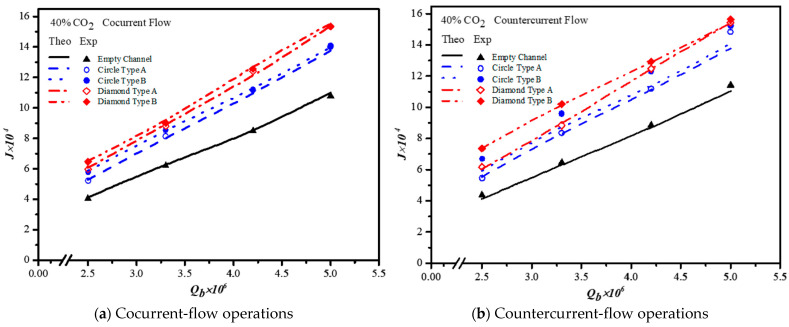
Effects of MEA flow rate and array configurations on absorption fluxes.

Embedding turbulence promoters plays a significant role in inducing a higher turbulence intensity to disrupt the mass-transfer boundary layer as well as to reduce mass-transfer resistance, which comes out the absorption flux improvement. The correlated Sherwood numbers indicate that the mass transfer coefficient of the module with embedding mini-channel Diamond turbulence promoters achieves a higher value than those of the modules using the empty channel and embedding Circle turbulence promoters, as shown in Figure 14a,b. The results showed that the module with inserting mini-channel Diamond turbulence promoters into flow channels boosts more intensive vortices and eddies due to a non-smooth curvature shape of obstacles than those in the module with inserting Circle turbulence promoters. Moreover, the correlated Sherwood numbers in countercurrent-flow operations are higher than those in cocurrent-flow operations. Good agreement was obtained in comparisons of both theoretical predictions and experimental results of the modules with embedding 3D printing mini-channel turbulence promoters, as demonstrated in Figure 15. The results showed that the CO_2_ absorption flux for the module with inserting 3D printing mini-channel turbulence promoter with both geometric shapes of Circle and Diamond turbulence promoters in both cocurrent- and countercurrent-flow operations, produces a larger turbulence intensity, and thus yields the higher mass transfer flux. Moreover, the CO_2_ permeates flux through the hydrophobic membrane in the module by embedding turbulence promoters and is more considerable in countercurrent-flow operations than that in concurrent-flow operations. 

**Figure 16:**
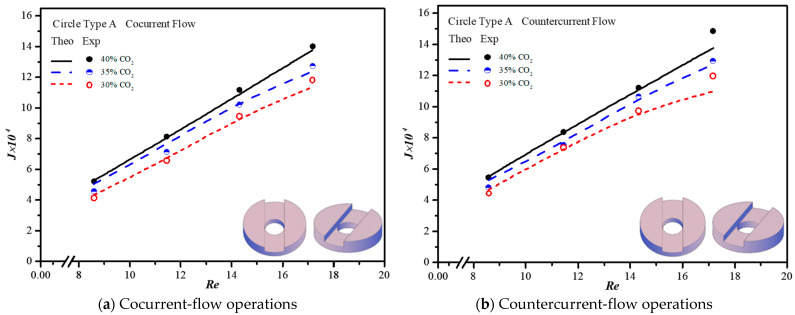
Effects of inlet CO_2_ concentrations with Circle promoters on the CO_2_ absorption flux.

**Figure 17:**
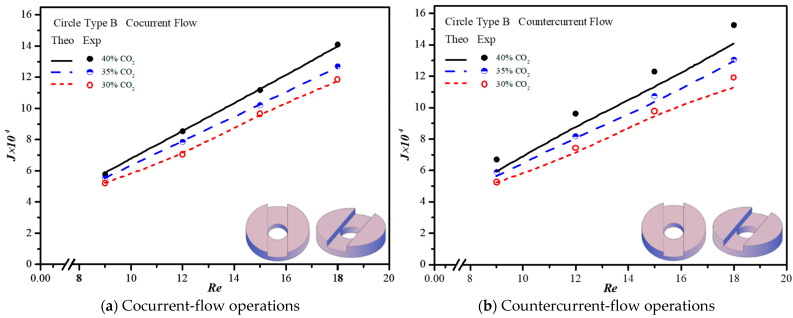
Effects of inlet CO_2_ concentrations with Circle promoters on the CO_2_ absorption flux.

**Figure 18:**
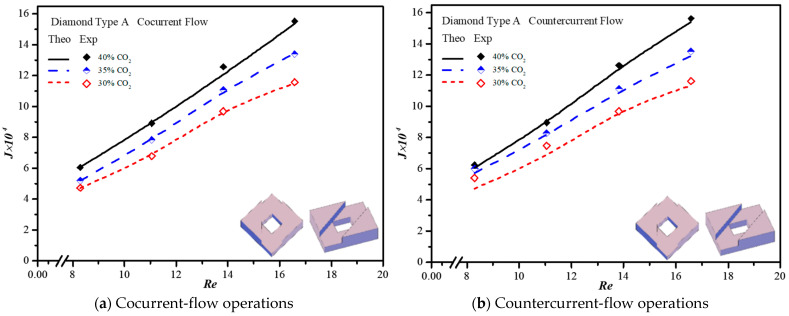
Effects of inlet CO_2_ concentrations with Diamond promoters on the CO_2_ absorption flux.

**Figure 19:**
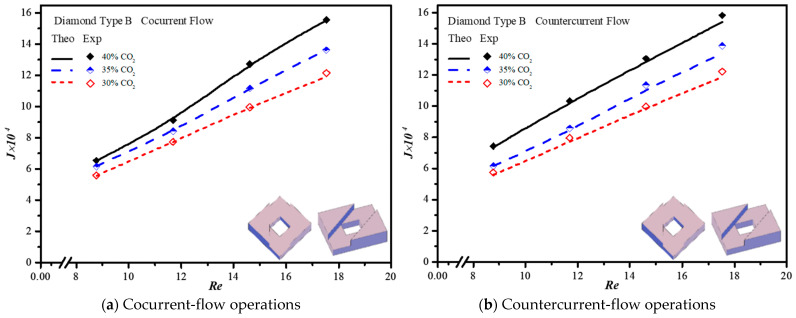
Effects of inlet CO_2_ concentrations with Diamond promoters on the CO_2_ absorption flux.

The CO_2_ absorption flux in the module with embedding 3D printing mini-channel turbulence promoters were presented graphically with the Reynolds number of the MEA feed rate, geometric shape, array configuration and flow pattern as parameters, as delineated in Figure 16, Figure 17, Figure 18 and Figure 19. The agreement of the theoretical results with those obtained from experimental results is apparently good. The extent of the CO_2_ absorption flux of both theoretical predictions and experimental results increases with the MEA feed flow rate and inlet feed CO_2_ concentration. The magnitude is in the order: Diamond Type B > Diamond Type A > Circle Type B > Circle Type A >  Circle Type A, as seen in Figure 16, Figure 17, Figure 18 and Figure 19. Embedding turbulence promoters play an important role in interrupting the concentration boundary layer by inducing a higher turbulence intensity on the membrane surface, and thus, the absorption flux improvement was boosted due to diminishing mass-transfer resistance. Two geometric shapes of turbulence promoters were prepared and compared for their absorption fluxes. As shown in Figure 16, Figure 17, Figure 18 and Figure 19, a higher absorption flux was achieved for inserting Diamond turbulence promoters. In the present study, inserting a non-smooth curvature geometric shape of Diamond turbulence promoters in the flow channels gave satisfactory-to-high absorption flux performance. The absorption flux improvement was also confirmed via operating various flow patterns and array configurations in the current study.

### 4.2. Absorption Flux Improvement and Further Absorption Flux Enhancement

**Figure 20:**
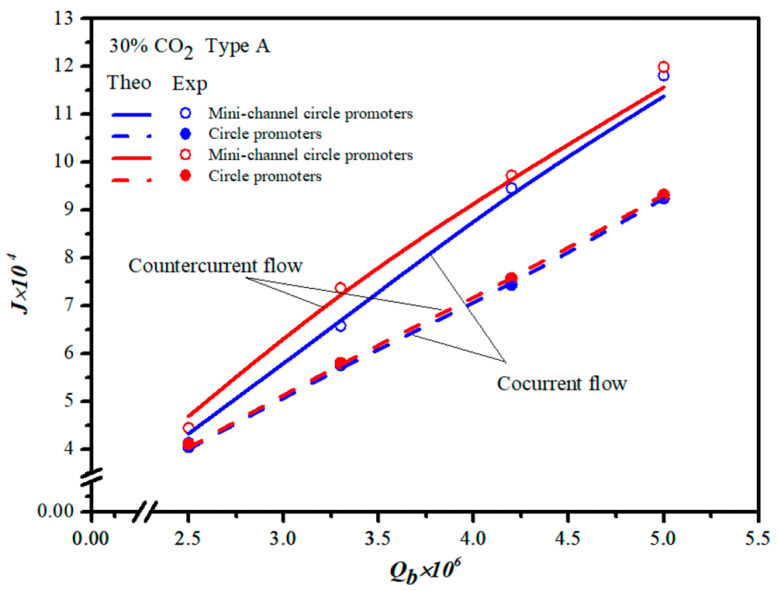
Comparisons of theoretical CO_2_ absorption flux of embedding 3D printing turbulence promoters with/without fabricating mini channels [43].

The present work extends the previous study except for embedding 3D printing mini-channel turbulence promoters instead of inserting turbulence promoters without fabricating mini-channels [43] for both concurrent- and countercurrent-flow operations, as shown in Figure 20. The present study illustrates why the present design of fabricating 3D mini-channel turbulence promoters is preferred regarding technical feasibility and comes out with a considerably larger absorption flux than that in our previous work [43].

Restated, a relative permeated flux improvement, say ${\;I}_{E},$ was evaluated by the percentage increase in the device with inserting 3D turbulence promoters, based on the device of the empty channel (wound with nylon fiber) under countercurrent-flow operations as an illustration, which are two kinds of turbulence promoters with inserting mini-channels (the present device) and without inserting mini-channels (the module used in Ref. [43]), respectively.

(20) $$I_{E,P}^{CT}\left(\%\right)=\frac{J_{E,p}^{CT}-J_{empty}^{CO}}{J_{empty}^{CO}}\times 100=\left(\frac{J_{E,p}^{CT}}{J_{empty}^{CO}}-1\right)\times 100,Module without mini-channel$$

(21) $$I_{E,MC}^{CO}(\%)=\frac{J_{E,MC}^{CO}-J_{empty}^{CO}}{J_{empty}^{CO}}\times 100,Module with mini-channel in cocurrent flow$$

(22) $$I_{E,MC}^{CT}(\%)=\frac{J_{E,MC}^{CT}-J_{empty}^{CO}}{J_{empty}^{CO}}\times 100,Module with mini-channel in countercurrent flow$$

where $I_{E,p}^{CT}$ and $I_{E,MC}^{CT}$ are the absorption flux improvement in the module of Ref. [43] and the module with embedding 3D printing mini-channel turbulence promoters for countercurrent-flow operations, respectively. Meanwhile, the subscripts *E* and *empty* denote the modules with/without embedding 3D turbulence promoters, respectively, while *MC* means the module with the mini-channel, and the superscripts *CO* and *CT* denote concurrent- and countercurrent-flow operations, respectively. Generally, the permeated flux augmented by inserting 3D turbulence promoters is more significant in countercurrent-flow operations than that in concurrent-flow operations. The further absorption flux enhancement $E_{p}$ of CO_2_ absorption in membrane contactors by embedding 3D Circle turbulence promoters is calculated based on the device of the same working dimensions performed in the previous work [43] under countercurrent-flow operations as follows:

(23) $$\begin{array}{ll} E_{P}(\%)=\frac{J_{E,MC}^{CT}-J_{E,P}^{CT}}{J_{E,P}^{CT}}\times 100= & \left[\frac{\left(J_{E,MC}^{CT}-J_{empty}^{CT})-(J_{E,P}^{CT}-J_{empty}^{CT}\right)}{J_{empty}^{CT}}\right]\left(\frac{J_{empty}^{CT}}{J_{E,P}^{CT}}\right)\times 100\\ & =\left(I_{E,MC}^{CT}-I_{E,P}^{CT}\right)/\left(1+I_{E,P}^{CT}\right)\times 100 \end{array}$$

where $J_{E,p}^{CT}$ and $J_{E,MC}^{CT}$ are the absorption flux in the module of Ref. [43] and the module with embedding 3D printing mini-channel turbulence promoters for countercurrent-flow operations, respectively. A percentage increment of absorption flux improvement and further absorption flux enhancement was evaluated for the module with embedding mini-channel turbulence promoter, which was compared to the absorption flux in the module by embedding turbulence promoter without the mini-channel for Circle turbulence promoters under two array configurations, respectively, as seen from Table 1.

The theoretical predictions show that the further absorption flux enhancement up to 30.56% is obtained with embedding Circle turbulence promoters of Type B array configurations, as demonstrated in Table 1. Generally, the further absorption flux enhancement of the module with embedding the mini-channel turbulence promoter decreases with the inlet feed CO_2_ concentration and the MEA feed flow rate. Meanwhile, a larger further absorption flux enhancement in operating the Circle turbulence promoter under Type B array configurations is achieved as compared to Type A array configurations at the lower MEA feed flow rate.

### 4.3. Power Consumption Increment

The power consumption increment is necessitated due to the increased frictional loss by embedding mini-channel turbulence promoters in the MEA absorbent feed stream of the parallel-plate gas/liquid membrane contactor modules, which were determined by only the friction losses to walls by using Fanning friction factor $f_{F}$ [49]:

(24) $$H_{i}=Q_{a}\;\rho_{CO{\;}_{2}}\;lw_{f,CO{\;}_{2}}+Q_{b}\;\rho_{MEA}lw_{f,MEA}i=promoter,\;empty$$

(25) $${lw}_{f,{CO}_{2}}=\frac{2f_{F,{CO}_{2}}\nu_{{CO}_{2}}^{2}L_{{CO}_{2}}}{D_{h,{CO}_{2}}}$$

(26) $${lw}_{f,MEA}=\frac{2f_{F,MEA}\nu_{MEA}^{2}L_{MEA}}{D_{h,MEA}}$$

in which the average velocity is calculated with the volumetric flow rate divided by the wetted area. The relative extents $I_{H}\;$ of the power consumption increment was illustrated based on the device of using the empty channel by calculating the percentage increment in the module with embedding 3D min-channel turbulence promoters as

(27) $$I_{H}=\frac{H_{promoter}-H_{empty}}{H_{empty}}\times 100\%$$

where the subscripts of *promoter* and *empty* represent the flow channels with and without embedding 3D printing mini-channel turbulence promoters, respectively.

**Figure 21:**
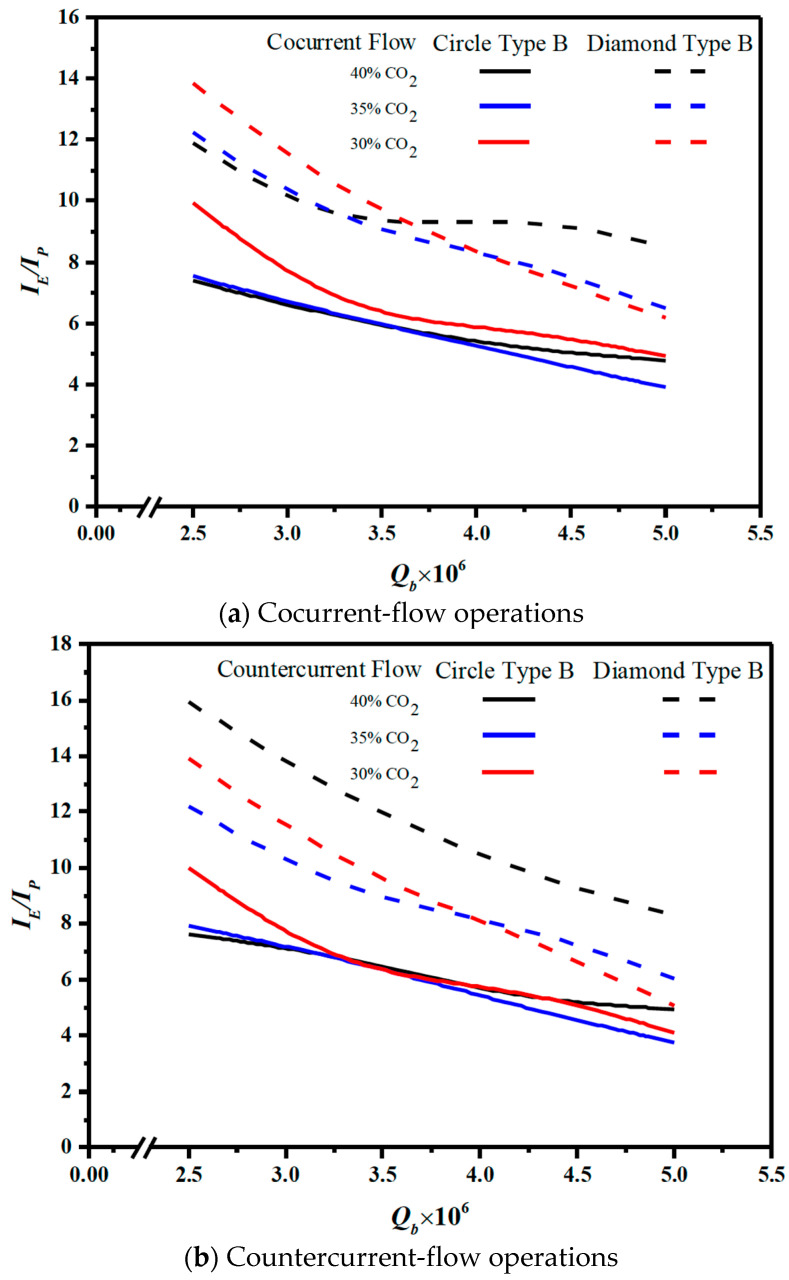
Effects of shapes of turbulence promoters with or without mini channels on $I_{E}/I_{P}$.

The efficacy of membrane turbulence promoters in terms of both desirable absorption flux improvement and the undesirable power consumption increment was assessed with an economic viewpoint for optimal operations, as referred to the ratio of $I_{E}/I_{H}$. Restated, utilizing turbulence promoters to diminish the concentration polarization effect could compensate the friction loss increment within a certain extent. Embedding 3D printing min-channel turbulence promoters in the MEA feed channel performed a better absorption flux improvement at the expense of a larger value of friction loss increment, which the effects on $I_{E}/I_{H}\;$ with geometric shapes of turbulence promoters, inlet feed CO_2_ concentrations, flow patterns and MEA flow rates as parameters are shown in Figure 21 with Type B array configuration as an illustration for the higher absorption flux improvement.

Figure 21 shows that the countercurrent-flow operations accomplish relatively larger $I_{E}/I_{H}$ values than those of the cocurrent-flow operations with respect to the economic consideration. Meanwhile, the order of the ratio of $I_{P}/I_{H}$ is expected with the same trend of the absorption fluxes with Diamond Type B > Circle Type B.

## 5. Conclusions

The designs of 3D printing min-channel turbulence promoters were applied to the membrane absorption module, which could swirl the flow stream so as to enhance the turbulence intensity in enhancing the mass transfer rate. The theoretical predictions and experimental results indicated that the device performance of embedding mini-channel turbulence promoters was boosted effectively owing to creating the secondary flow pattern and augmenting the turbulence intensity. The present study serves as a groundwork investigation of the important findings for utilizing min-channel turbulence promoters for MD application. The conclusions are drawn in this proof-of-design study as follows: 

(a) Operating MD module by embedding 3D printing mini-channel turbulence promoters with various geometric promoter-shapes and array configurations resulted in enhanced absorption flux performance in comparison with the module with using the empty channel due to generating vortexes and eddies.

(b) The theoretical predictions show that the further absorption flux enhancement up to 30.56% is obtained with embedding Circle mini-channel turbulence promoters of Type B array configurations as compared to that in the module without inserting mini-channel turbulence promoters. Meanwhile, the higher absorption flux improvement is obtained by embedding turbulence promoters of Type B configuration compared to Type A configuration.

(c) The improved absorption fluxes by embedding mini-channel turbulence promoters were augmented and represented with a simplified expression of the correlated Sherwood number. The correlated Sherwood numbers obtained in the module with embedding 3D mini-channel Diamond turbulence promoters achieved a higher value than those of the devices with the empty channel and embedding Circle turbulence promoters. Moreover, the correlated Sherwood numbers in the module under countercurrent-flow operations are higher than those in operating cocurrent-flow patterns.

(d) The power consumption increment was increased due to embedding the 3D mini-channel turbulence promoter to cause a drop in fluid pressure. The economic viewpoint was examined in terms of the ratio of the absorption flux improvement to power the consumption increment, say $I_{E}/I_{P}$. The results indicated that the ratio $I_{E}/I_{P}$ for Type B configuration is higher than that of Type A configuration.

The present study only explores two specific geometric shapes and two array configurations under the specific dimension designs. Embedding 3D printing min-channel turbulence promoters to the gas/liquid membrane absorption module with the use of MEA absorbent shows a great potential to considerably enhance the absorption flux. A new design in this study includes the advantage effect of reinforcing the turbulence intensity as an alternative tactic on the absorption flux in the membrane absorption module with embedding 3D mini-channel turbulence promoters. However, there still exists many more possibilities to other designs of mini-channel turbulence promoters in finding an optimal device performance with considering the economic viewpoint for membrane absorption processes. 

## Figures and Tables

**Table 1:** Comparisons between both absorption flux improvements $E_{P}(\%)$.

$C_{in}q_{b}\times 10^{6}$ (%) (m^3^/s)		Countercurrent-Flow Operations					
		Circle (Ref. [45])		Circle [The present work]			
		Type A	Type B	Type A			Type B
		$I_{E,P}^{CT}(\%)$	$I_{E,P}^{CT}(\%)$	$I_{E,MC}^{CT}(\%)$	$E_{P}(\%)$	$I_{E,MC}^{CT}(\%)$	$E_{P}(\%)$
30	5.0	21.88	23.62	44.19	18.30	61.40	30.56
	6.67	20.11	23.14	43.91	19.82	38.06	12.17
	8.33	19.68	22.59	36.47	14.03	35.46	13.19
	10.0	17.52	19.02	28.37	9.23	25.37	5.34
35	5.0	24.58	25.42	39.85	12.26	48.73	18.59
	6.67	22.92	24.09	34.47	9.40	41.79	14.26
	8.33	21.35	22.95	34.80	11.08	31.07	6.61
	10.0	21.06	22.67	24.92	3.19	23.12	3.67
40	5.0	26.70	32.04	37.16	8.26	46.95	11.29
	6.67	25.81	31.45	34.86	7.19	42.29	8.25
	8.33	22.91	29.80	30.05	5.81	32.53	2.10
	10.0	21.94	28.86	27.86	4.86	30.60	1.35

## Data Availability

Data is contained within the article.

## Abbreviations

A	Wetted area (m^2^)
C	Concentration (mol m^−3^)
$C_{mean}$	Mean value of C (mol m^−3^)
$c_{k}$	Membrane coefficient based on the Knudsen diffusion model ($\mathrm{mol}\;m^{-2}Pa^{-1}s^{-1}$)
$c_{M}$	Membrane coefficient based on the molecular diffusion model ($\mathrm{mol}\;m^{-2}Pa^{-1}s^{-1}$)
$c_{m}$	Membrane permeation coefficient ($\mathrm{mol}\;m^{-2}Pa^{-1}s^{-1}$)
$D_{h,i}$	Equivalent hydraulic diameter of channel (m), i=promoter empty
$E_{P}$	Further absorption flux enhancement
Er	Accuracy deviation of experimental results from the theoretical predictions
$f_{F}$	Fanning friction factor
$H_{C}$	Dimensionless Henry’s constant
H	Channel height (m)
$H_{i}$	Hydraulic dissipate energy (J kg^−1^), i=carbon, empty
$I_{E}$	Absorption flux enhancement
$I_{H}$	Power consumption relative index
J	Absorption flux (mol m^−2^ s^−1^)
$k_{a}$	Mass transfer coefficient in the CO_2_/N_2_ stream (m s^−1^)
$k_{b}$	Mass transfer coefficient in the MEA absorbent stream (m s^−1^)
$K_{ex}$	Equilibrium constant
$K_{ex}^{\prime }$	Reduced equilibrium constant
$K_{m}$	Overall mass transfer coefficient of membrane (m s^−1^)
$lw_{f,j}$	Friction loss (J kg^−1^), $\;j=CO_{2},MEA$
L	Channel length (m)
$M_{W}$	Molecular weight of water (kg mol^−1^)
*N_exp_*	Number of experimental measurements
P	Wetted perimeter (m)
$P_{1}$	Saturation vapor pressure in the CO_2_/N_2_ stream (Pa)
$P_{2}$	Saturation vapor pressure in the MEA absorbent stream (Pa)
$Q_{a}$	Volumetric flow rate of the gas feed stream (m^3^ s^−1^)
$Q_{b}$	Volumetric flow rate of the MEA absorbent side (m^3^ s^−1^)
R	Gas constant (8.314 J mol^−1^ K^−1^)
*Re*	Reynolds number
${Sh}_{promoter}$	Enhanced dimensionless Sherwood number
${Sh}_{lam}$	Sherwood number for laminar flow
$W_{1}$	Average width of the promoter (m)
$W_{2}$	The punched hole diameter (m)
$W_{3}$	The average width of the punched hole inside the promoter (m)
${\left|Y_{m}\right|}_{ln}$	Natural log mean CO_2_ mole fraction in the membrane
z	Axial coordinate along the flow direction (m)
Greek letters	
$\alpha^{E}$	Enhancement factor
$\delta_{m}$	Thickness of membrane (µm)
ε	Membrane porosity
$\overset{-}{\nu }$	Average velocity (m^3^ s^−1^)
$\rho_{i}$	Density (Kg m^−3^),$\;i=CO_{2},\;MEA$
$\gamma_{m}$	Concentration polarization coefficients
Subscripts	
1	Membrane surface on gas feed side
2(l)	Membrane surface on MEA side
2 ( g)	Membrane surface on CO_2_/N_2_ side
*a*	CO_2_/N_2_ feed flow channel
*b*	MEA absorbent flow channel
*cal*	Calculated results
*empty*	Channel without embedding turbulence promoters
*exp*	Experimental results
*g*	CO_2_/N_2_ feed side
*in*	At the inlet
l	MEA feed side
*out*	At the outlet
*promoter*	Channel embedding 3D mini-channel turbulence promoters
*theo*	Theoretical predictions
